# Antithrombin III is probably not a suitable biomarker for diagnosis of primary central nervous system lymphoma

**DOI:** 10.1007/s00277-015-2334-y

**Published:** 2015-02-21

**Authors:** Milla Elvi Linnea Kuusisto, Kirsi-Maria Haapasaari, Anne Marja Remes, Risto Bloigu, Peeter Karihtala, Taina Turpeenniemi-Hujanen, Outi Kuittinen

**Affiliations:** 1 Department of Oncology and Radiotherapy, University of Oulu, Medical Research Center, and Oulu University Hospital, Kajaanintie 50, 90220 Oulu, Finland; 2 Department of Pathology, University of Oulu and Oulu University Hospital, PL50, 90029 OYS Oulu, Finland; 3 Institute of Clinical Medicine–Neurology, University of Eastern Finland and Department of Neurology, Kuopio University Hospital, P.O. Box 1627, 70211 Kuopio, Finland; 4 Medical Informatics and Statistics Research Group, University of Oulu, Aapistie 7, P.O. Box 5000, 90014 Oulu, Finland

**Keywords:** Antithrombin III, PCNSL, Lymphoma, CSF

## Abstract

Antithrombin III (AT III) in cerebrospinal fluid (CSF) has been suggested to have high specificity and sensitivity in separating primary central nervous system (CNS) lymphoma from other neurological conditions. We measured with ELISA CSF and serum AT III and albumin levels in 12 lymphoma patients with CNS involvement, 30 lymphoma patients without CNS involvement, and 41 patients with non-neoplastic neurological diseases. AT III immunostaining was also carried out, in lymphoma patients. Both CSF AT III and albumin levels were higher in lymphoma patients with CNS involvement. AT III/albumin ratio in CSF was the most sensitive and specific measure for diagnosis. Lowest it was in patients with known CNS lymphoma. Serum AT III levels were lower both in CNS lymphoma and systemic lymphoma. CSF AT III levels were shown to be higher in lymphoma patients with CNS involvement, when AT III/albumin ratios were lower. This was probably a result of lowered serum AT III levels, indicating that high levels of AT III in CSF might reflect only leakage of the blood–brain barrier. Thus, AT III fails to be a specific marker for diagnosis of lymphoma CNS involvement.

## Introduction

About 2 % of all systemic lymphomas are primary central nervous system (CNS) lymphomas (PCNSLs). These are rapidly progressing diseases with often fatal outcome despite the use of high-dose methotrexate-containing treatment regimens [1]. Usually, the diagnosis of PCNSL is based on a histological sample taken by stereotactic biopsy. However, sometimes in the early stage of the disease, there is no radiologically apparent target tissue for the biopsy or the disease may mimic another neurological disorders. Cerebrospinal fluid (CSF) has also been used in the diagnostic and differential process, although the associated cytology is not sensitive and the results can be negative in up to 60 % of the patients [2, 3]. The disease is rapidly progressing; there is a need for urgent diagnostic work-up and start of therapy. There is also a need for sensitive and specific biomarkers for early diagnosis.

In addition to the above, lymphomas presenting primarily as systemic disease may recur in the CNS. The prognosis in cases of CNS relapse is dismal. At least 5 % of patients with diffuse large B cell lymphoma (DLBCL) will develop CNS involvement if appropriate prophylactic therapy is not given [2]. Early CNS relapses probably arise from subclinical CNS disease already present at the time of the primary diagnosis. Prophylactic therapies cannot be given to all patients with systemic lymphoma as a result of the high toxicity rate. There is an unmet clinical need for good biomarkers for selection of DLBCL patients with subclinical CNS involvement for prophylactic therapies.

Antithrombin III (AT III) is a protein produced by hepatocytes, and its physiological role is to play a part in the normal anticoagulation system [4–6]. AT III has also been detected in CSF [6–9].

It was suggested in a paper by Roy et al. that AT III in CSF is a specific biomarker of PCNSL, and more accurate in identification of CNS lymphoma than CSF cytology [7, 8]. The author also suggested that AT III level alone is sufficient for a diagnosis of PCNSL. However, increased concentrations of AT III in CSF might merely reflect leakage of the blood–brain barrier [9].

The aim of the present study was to evaluate whether or not CSF AT III is an independent biomarker of lymphoma CNS lesions or a marker of blood–brain barrier leakage, and whether it could be used to find patients with systemic lymphoma and subclinical CNS disease. The biology of AT III was also explored to see if it is present in endothelial and/or lymphoma cells, to discover the possible origin of CSF AT III.

## Materials and methods

### Patients and sample collection

The lymphoma study samples were taken from patients treated at Oulu University Hospital during 2009–2012 after receiving written informed consent in each case. All patients suffered from histologically confirmed lymphoma. The clinical data was collected carefully from hospital records. The principles of Declaration of Helsinki were followed in this study. Ethical resolution was applied from the Local Ethics Committee of the Northern Ostrobothnia Hospital District and from National Supervisory Authority for Welfare and Health (Valvira). Valvira permission number was 6622/05.01.00.06/2010.

Patient details are presented in Table 1. The patients were selected for this study because of the availability of their CSF, which was taken during normal lymphoma diagnostic and therapeutic procedures. Samples from patients without CNS disease were taken while treating them by means of routine prophylactic intrathecal therapy. Prophylactic therapy included administration of high-dose methotrexate given intravenously and intrathecally, or simply intrathecally. The prophylaxis was given to patients with International Prognostic Index of 3–5 and to patients with testicular, paraspinal, or breast location of lymphoma. One of the patients in the present study was given intrathecal methotrexate and four were given both intravenous and intrathecal methotrexate before CSF samples were taken. Assays of CSF and serum albumin concentrations were performed as part of normal laboratory routines. For CSF AT III assay, only samples taken via lumbar puncture were included. Macroscopically bloody samples were excluded. Standardization of sample taking was ensured by using only one physician for the procedure. Three cases were excluded because CSF was taken via a reservoir used for intrathecal therapy. The serum samples from these patients, however, were included in the evaluation. Matched CSF and serum samples were available in 15 cases. Altogether, 42 CSF samples and 18 serum samples were available for analysis. Immunohistochemical analysis was carried out using lymphoma tissue samples from the same patients.

**Table 1:** Demographics of the patients

	Patients with systemic lymphoma and CNS lesion		Patients with systemic lymphoma without CNS lesion		Patients with PCNSL	
	(*n* = 7)		(*n* = 29)		(*n* = 5)	
Age						
Median (years)	71		57		62	
Range (years)	70–79		18–81		50–75	
Sex						
Male	5	(71.4 %)	13	(44.8 %)	3	(60 %)
Female	2	(28.6 %)	16	(55.2 %)	2	(40 %)
Lymphoma subtype						
DLBCL	6	(85.7 %)	16	(55.2 %)	2	(40 %)
Mantle cell lymphoma	1	(14.3 %)	0		0	
Atypical Burkitt’s lymphoma	0		8	(27.6 %)	2	(40 %)
Burkitt’s lymphoma	0		1	(3.4 %)	0	
T-cell-rich B cell lymphoma	0		1	(3.4 %)	0	
Follicular lymphoma	0		2	(6.9 %)	1	(20 %)
Lymphocytic lymphoma	0		1	(3.4 %)	0	
	Patients with Alzheimer’s disease		Patients with multiple sclerosis		Patients with no neurological illness	
	(*n* = 19)		(*n* = 7)		(*n* = 37)	
Age						
Median (years)	61		39		49	
Range (years)	51–70		31–46		26–73	
Sex						
Male	9	(47.4 %)	3	(42.9 %)	20	(54.1 %)
Female	10	(52.6 %)	4	(57.1 %)	17	(45.9 %)

*PCNSL* primary central nervous system lymphoma, *DLBCL* diffuse large B cell lymphoma

As a control group, 42 CSF samples were collected from patients with non-neoplastic neurological diseases (all taken by lumbar puncture). These were 19 cases with clinically, genetically, or autopsy-confirmed Alzheimer’s disease, 6 patients with multiple sclerosis (MS) diagnosed according to McDonald’s 2005 criteria [10] and also with MS-typical CSF findings, and 17 cases who suffered from subjective memory problems connected with depression without any signs of progressive neurodegenerative disease or neurological condition within 3 years of follow-up. Serum samples from these cases were not available. These samples were collected at the Department of Neurology, Kuopio University Hospital during normal diagnostic procedures. Patient details are shown in Table 1. Twenty serum samples taken from healthy controls were also available.

### ELISAs

Antithrombin III concentrations in CSF were detected by using an ELISA. Commercial assay kits (Human Antithrombin III ELISA kit, cat. No. KSP-110, Nordic BioSite, Täby, Sweden) were used. The principles of this assay have been described previously [11], and kits were used according to the manufacturer’s instructions. The method is based on a quantitative enzyme immunoassay technique. In this assay, AT III in standards and samples is sandwiched between immobilized antibody and biotinylated polyclonal antibody specific for AT III, and the complex is recognized by a streptavidin-peroxidase conjugate.

Blood was collected in siliconized 3.5-ml glass containers containing one part serum gel solution. Serum was prepared from blood by centrifugation at 1500 *× g* for 10 min. AT III concentrations were measured by ELISA (Human Antithrombin III ELISA kit, cat. no. KSP-111, Nordic BioSite, Täby, Sweden) according to the manufacturer’s instructions. In this assay, standards and samples are competed with a biotinylated AT III sandwiched by the immobilized antibody and streptavidin-peroxidase conjugate.

### Immunohistochemistry

For immunohistochemical analyses, 42 tissue samples were available. Most of them were lymph node biopsy samples, but brain and ventricle biopsy samples were also present (lymph nodes, *n* = 38; brain, *n* = 3; ventricle, *n* = 1). All tissue samples had been surgically removed from patients during normal diagnostic procedures and used for the study later. Immunostaining was performed as described previously [12], with a few modifications: Novolink kits were used according to the manufacturer’s instructions. Antibody dilution was 1:35. Samples were microwaved with Tris-EDTA, pH 9, and incubated at room temperature for 1 h before immunostaining with a commercial polyclonal antithrombin III antibody (Novus Biologicals, Littleton, USA).

### Statistical analysis

Statistical analysis was performed using SPSS Data Editor Software version 20 (IBM Corp. Released 2011. IBM SPSS Statistics for Windows, version 20.0. Armonk, NY). Continuous variables with normal distributions were compared by using independent samples *t* tests. Serum AT III and albumin concentrations showed normal distributions. Continuous variables with skewed distributions were compared by using the Mann–Whitney *U* test. Because CSF AT III and albumin concentrations did not show normal distributions, median values were used for comparison. Values of *p* < 0.05 were considered to be statistically significant.

## Results

The median age of the patients with lymphoma (21 males, 21 females) was 62.5. The non-lymphoma patients (32 males, 31 females) were somewhat younger (median age 52).

### AT III and albumin concentrations in cerebrospinal fluid

**Fig. 1:**
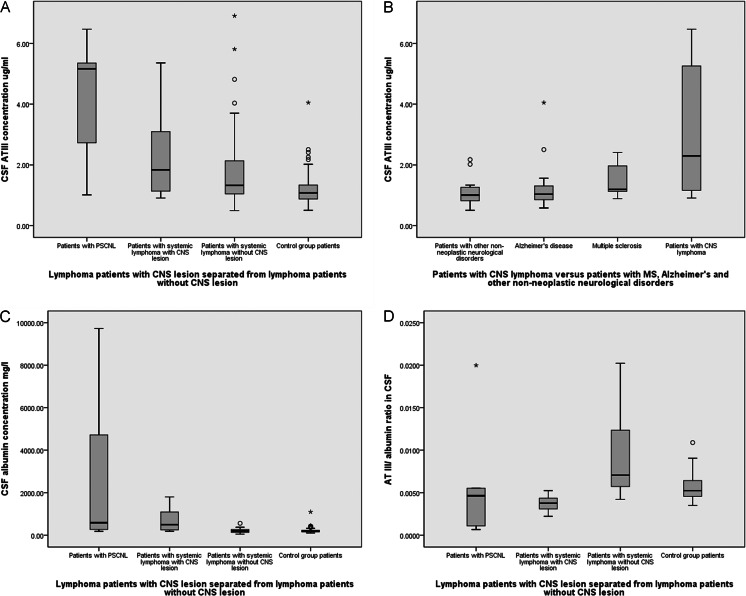
Cerebrospinal fluid (CSF) antithrombin III (AT III) and albumin concentrations and AT III/ albumin concentration ratio in different patient groups. **a** AT III concentrations in patients with primary CNS lymphoma (PCNSL), secondary CNSL (sCNSL), systemic lymphoma, and non-neoplastic disease as a control group of patients. **b** AT III concentrations in patients with CNS lymphoma (both PCNSL and sCNSL), multiple sclerosis (MS) disease, Alzheimer’s disease, and in control group patients. **c** Albumin concentrations in patients with PCNSL, sCNSL, systemic lymphoma without CNS lesion, and non-neoplastic disease as a control group of patients. **d** AT III/albumin ratio in patients with PCNSL, sCNSL, systemic lymphoma without CNS lesion, and non-neoplastic disease as a control group of patients

The median CSF AT III concentration was found to be 1.47 μg/ml (range 0.49–6.91) in the lymphoma patients, and it was 1.08 μg/ml (0.50–4.05) in the group of patients with non-neoplastic neurological disorders (Table 2). The difference between these two groups was statistically highly significant (*p* = 0.002, in Mann–Whitney *U* test). The median albumin concentration in the CSF of patients with lymphoma was 243 mg/l (52.5–9727), and it was 193 mg/l (105.5–1099) in the non-lymphoma group. This difference was not statistically significant (*p* = 0.219). Concentrations of CSF AT III were also compared in different patient groups (Fig. 1a). When comparing CSF AT III concentrations in patients with PCNSL with those in patients with secondary CNS lymphoma or lymphoma patients without CNS lesions, or with the non-lymphoma group of patients, a statistically significant difference was seen indicating that CSF AT III concentrations were highest in the group of PCNSL patients (*p* = 0.006). Concentrations of CSF AT III were also compared in patients with CNS lymphoma (including PCNSL patients and patients with CNS-relapsed systemic lymphoma) versus those with non-neoplastic neurological diseases. In this comparison, in patients with CNS lymphoma, CSF AT III concentrations remained still higher (*p* = 0.009), as seen in Fig. 1b. Similar differences were found when the same groups were compared to CSF albumin concentrations, although in patient group of PCNSL, albumin concentrations were much higher than in other patient groups (Fig. 1c, *p* = 0.006). We compared CSF AT III concentrations in patients with MS disease with those in lymphoma patients with CNS involvement (both primary and secondary), and the concentrations were rather similar (*p* = 0.167). When comparing CSF AT III concentrations between groups of lymphoma patients with CNS involvement (both primary and secondary) versus lymphoma patients with systemic disease only, no differences were found (*p* = 0.126).

**Table 2:** Cerebrospinal fluid (CSF) and serum antithrombin III (AT III) and albumin levels

	Patients with lymphoma			Non-lymphoma patients			
	(*n* = 42)			(*n* = 61)			
Characteristic	Median	Number	Range	Median	Number	Range	*p*
Age (years)	62.50	42	18–81	52	61	26–70	0.001^a^
CSF AT III (μg/ml)	1.471	42	0.49–6.91	1.077	41	0.50–4.05	0.002^a^
CSF albumin (mg/l)	242.82	42	52.51–9727.70	192.77	41	105.52–1099.02	0.219^a^
Serum AT III (mg/ml)	0.0213	18	0.01–0.03	0.0296	20	0.02–0.04	0.001^b^
Serum albumin (g/l)	40.944	18	26–49	44.55	20	37–49	0.029^b^

^a^Mann–Whitney *U* test

^b^Independent samples *t* test

The AT III/ albumin ratio was calculated as reported previously [9]. When comparing this ratio in the different groups of patients with PCNSL, secondary CNS lymphoma, systematic lymphoma without CNS involvement, and non-lymphoma patients (Fig. 1d), patients with systemic lymphoma without CNS lesion had the highest AT III/albumin ratio (*p* < 0.001).

### AT III and albumin concentrations in serum

Antithrombin III and albumin concentrations were also assayed in serum, as shown in Table 2. The mean serum AT III concentration in patients with lymphoma was 0.021 mg/ml (range 0.01–0.03) and 0.03 mg/ml (0.02–0.04) in the non-lymphoma group. The difference between these two groups was statistically significant (*p* < 0.001). The mean serum albumin level was 40.94 g/l (26–49) in patients with lymphoma and 44.55 g/l (37–49) in the non-lymphoma group. This difference was also statistically significant (*p* = 0.029).

### Correlations between AT III immunostaining and serum and CSF AT III levels

**Fig. 2:**
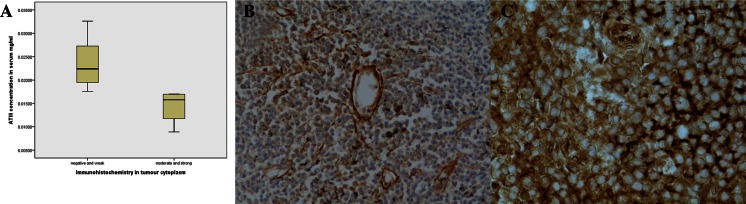
**a** Tumor cell antithrombin III (AT III) immunoreactivity reverse correlation with serum AT III concentrations. **b** Immunohistochemistry of lymphoma tissue, demonstrating tumor cell cytoplasmic expression of AT III and also AT III staining positivity around capillaries. Antithrombin III antibody staining, ×40 magnification. **c** Immunohistochemistry of lymphoma tissue, demonstrating strong expression of AT III in tumor cell cytoplasm and nuclear membrane. Antithrombin III antibody staining, ×100 magnification

The amount of immunoreactive protein staining was analyzed in three separate groups; tumor cytoplasm, tumor nuclear membrane, and tumor vasculature. In Fig. 2a, the immunohistochemical (IHC) results are compared with respective CSF AT III concentrations. Moderate and strong expression of cytoplasmic AT III in lymphoma cells (Fig. 2b) correlated with high concentrations of AT III in the CSF. The same kind of a trend was also seen as regards the nuclear membrane expression of AT III. However, endothelial expression in tumor vasculature did not correlate with AT III concentrations in the CSF. Serum AT III levels were also compared with IHC results. Lower AT III concentrations in the serum correlated with moderate and strong expression of AT III both in tumor cytoplasm and on nuclear membranes (*p* = 0.036 and *p* = 0.037, Fig. 2c). There was no statistically significant correlation when comparing the AT III levels with staining in tumor vasculature.

### Receiver operating characteristic curves

**Fig. 3:**
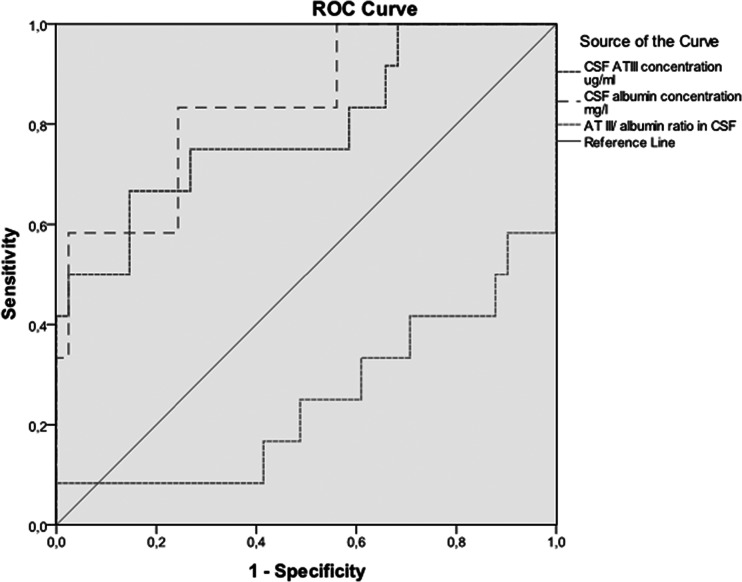
Receiver operating characteristic (ROC) curve analysis of CNS lymphoma patients, including both primary and secondary CNS lymphomas, versus patients with non-neoplastic neurological disorders to evaluate the possible role of antithrombin III (AT III) in cerebrospinal fluid (CSF) to be a novel biomarker to separate CNS lymphomas from other neurological disorders. 0-hypothesis was area under curve (AUC) 0.500, and values close to 1.000 are found significant and possibly useful as biomarkers in ROC analysis

We evaluated receiver operating characteristic (ROC) curves of CNS lymphoma patients, including both primary and secondary CNS lymphomas, versus patients with non-neoplastic neurological disorders. All diagnostic tests yielded somewhat similar results: CSF AT III/albumin ratio had area under curve (AUC) 0.250 (*p* = 0.009), CSF AT III alone had AUC 0.791 (*p* = 0.002), and CSF albumin had AUC 0.839 (*p* < 0.001) (Fig. 3). 0-hypothesis was AUC 0.500, and values close to 1.000 are found significant and possibly useful as biomarkers in ROC analysis.

## Discussion

In the present study, we found that CSF AT III concentrations were higher in patients with CNS lymphoma compared with patients with non-neoplastic neurological diseases. However, the values overlapped with each other, indicating that the specificity of CSF AT III measurement for CNS lymphoma is not valid for clinical use. Moreover, high CSF albumin levels had better specificity for CNS lymphoma than did CSF AT III levels. The most specific test to separate groups with lymphoma CNS involvement from other patient groups was the CSF AT III/ albumin ratio. Surprisingly, low ratios predicted the existence of primary or secondary CNS lymphoma. To study further the reason for these low ratio levels, we also measured the serum AT III concentrations. We found low serum AT III levels in lymphoma patients, which might explain the low CSF AT III/ albumin ratio in these patients. In MS in which the blood–brain barrier is probably leaking [13], the concentrations of AT III in the CSF were only slightly higher than in other neurological groups. There was no statistically significant difference when comparing CSF AT III concentrations in MS patients with those in lymphoma patients with CNS involvement (*p* = 0.167). Taken together, these findings indicate that CSF AT III might reflect only a blood–brain barrier leakage, and it may not be an independent marker of CNS lymphoma.

Antithrombin III is a physiological anticoagulative protein produced by hepatocytes [6]. It is involved both in intravascular and extravascular coagulation, and it is present normally in blood and lymph both in soluble form and bonded to thrombin-forming reversible AT III–thrombin complexes [6, 14]. AT III immunoreactive protein has been found previously in lymphoma cells [7]. Similar findings were also seen in the present study in lymphoma tissue samples both from brain and other tissues. Rubenstein et al. [7, 8, 15] evaluated AT III production in diagnostic PCNSL samples by gene expression profiling and suggested that lymphoma cells could produce the AT III. Due to the diffuse spread of malignant lymphocytes in the CNS, it is impossible, however, to specify exactly from which cells the AT III might be derived originally, and the knowledge of AT III biology in CSF is also so far incomplete [16]. Moreover, there are reports showing that AT III can detach from endothelial cells. This might be the best explanation for AT III positivity in the CNS [17].

There have been several studies on cancer patients in which changes in serum AT III levels have been investigated, with variable results [5, 6, 18, 19]. In studies including patients with lymphoma, lowered serum AT III concentrations have been discovered in active disease [18]. Successful treatment seems to normalize values, but there are still statistically significant differences between groups of lymphoma patients versus controls [18].

In our study, we detected lowered serum AT III concentrations in lymphoma patients, which may be caused by either increased use or decreased production [18]. We also found a correlation between lowered serum AT III concentrations and moderate and strong cytoplasmic and nuclear membrane AT III positivity in immunohistochemistry, suggesting that serum AT III concentrations decrease while lymphocytes accumulate AT III. Because there is elevated thrombin production in cancer patients, there could also be elevated consumption of AT III in serum. In addition, the fact that AT III can detach from endothelial cells and then bond with soluble thrombin [17] could indicate elevated consumption of AT III in lymphoma patients similarly as found in other cancer patients.

In a previous study by Roy et al., a mean CSF AT III level of 3.1 μg/ml was reported in CNS lymphoma patients and a mean value of 0.53 μg/ml in control patients without lymphoma [7]. In the present study, concentrations in the CSF did not follow a normal distribution and median values were used for evaluation. The median concentrations among our patients were 1.5 μg/ml (range 0.49–6.91) in those with lymphoma and 1.1 μg/ml (0.50–4.05) in the control group patients. The difference was statistically significant but the values overlapped. However, the difference between lymphoma patients with and without known CNS disease was also statistically significant, indicating that in patients with known CNS lymphoma diagnosis, CSF AT III concentrations were higher (*p* = 0.006). It has been previously suggested that CSF AT III concentrations could allow differentiation of CNS lymphoma from other malignant CNS conditions [7]. This claim was based on an AUC value of AT III in CSF. We evaluated here the AUC values for AT III, albumin, and the AT III/albumin ratio. The difference between AT III (AUC = 0.791, *p* = 0.002) and albumin (AUC = 0.839, *p* < 0.001) was not clear, indicating that AT III is not a novel biomarker that could be used to select patients with lymphoma from those with other diseases.

The patients in this study were treated with CNS prophylactic therapy due to a high risk of CNS relapse, and there was also a possibility that some of them primarily had subclinical CNS disease. This might have influenced the results concerning AT III concentrations in the CSF. The median age of the patients was also higher than in the non-lymphoma patients, which might have had an effect on the results. There was no evaluation of neurodegenerative diseases in the lymphoma patients. Serum samples were not available from the patients with neurological conditions, but it is not likely that this would have had an impact on the results.

The difference between the present study and the previous ones [7, 8] is that we used both PCNSL patients and systemic lymphoma patients without CNS involvement as a lymphoma group. Despite the fact that in these cases lymphoma affected brain or nodal areas only, it seems that CSF AT III concentrations are higher in cases of lymphoma. The biology of the process needs further study. It might be that CSF AT III is involved in inflammatory conditions [16].

In summary, in the present study, we found that elevated CSF AT III levels in patients with CNS involvement of lymphoma reflect the magnitude of blood–brain barrier disruption and AT III is not an independent biomarker of lymphoma CNS involvement.
